# From Digital Commons to Alma/Primo: enhancing the dissemination of Doctor of Nursing Practice (DNP) projects

**DOI:** 10.5195/jmla.2023.1624

**Published:** 2023-10-02

**Authors:** Sara Hoover

**Affiliations:** 1 shoover@gwu.edu, Metadata and Scholarly Publishing Librarian, Himmelfarb Health Sciences Library, The George Washington University, Washington, DC.

**Keywords:** Institutional Repositories, Metadata

## Abstract

The Doctor of Nursing Practice (DNP) project collection is a group of approximately 120 DNP projects archived between 2017 and 2022 in Health Sciences Research Commons (HSRC), the health sciences institutional repository (IR) for the George Washington University. Our project focused on expanding avenues for the dissemination of DNP projects beyond our Digital Commons IR by integrating this content into the library's instances of Ex Libris Alma and Primo VE. By utilizing the Ex Libris Repository type import profile rather than the OAI-PMH feed, we identified enhanced opportunities for content discovery while likewise retaining better control over individual item records.

## PROJECT BACKGROUND

The George Washington University (GW) is an R1 research institution located in Washington D.C. and the Himmelfarb Health Sciences Library is one of several GW libraries. Himmelfarb has maintained an instance of Digital Commons (Health Sciences Research Commons [1] or HSRC) since 2012 as an institutional repository (IR) with the goal of providing broad access to institutional health sciences related research. In 2018 Himmelfarb joined the Washington Research Library Consortium (WRLC) and migrated to Ex Libris's library services platform Alma and its discovery service Primo VE. Alma allows content from Open Archives Initiative Protocol for Metadata Harvesting (OAI-PMH) compliant archives to be loaded to Primo VE for discovery and librarians at Himmelfarb were eager to integrate content from the IR into Alma/Primo VE. Post-migration an OAI feed was established between HSRC and the Himmelfarb instance of Primo VE, but the project team immediately identified problems with adding all IR content to the library catalog. Citation only IR entries linking to the library catalog made for duplicative content and loading records directly into Primo's Central Discovery Index (CDI) prevented manual editing. The OAI feed was ultimately disable and the project team sought alternatives that could allow them to integrate more selective IR content.

## PROJECT FOCUS: DISSEMINATING DOCTOR OF NURSING PRACTICE PROJECTS

Although the rise of search engines has led to the diminishing role of discovery services within academic libraries [2], the library catalog nevertheless still offers a potentially important platform for discovery. A growing body of literature highlights the importance of grey literature in health sciences related research [3-5] and the inclusion of institution specific grey literature in a discovery system can help to advance the dissemination of quality, institution specific research. This has the benefit of helping researchers to identify possible collaborators and to potentially address practice gaps that can improve patient safety [6]. The project team began a content integration project by focusing on projects from the Himmelfarb IR Doctor of Nursing Practice (DNP) project collection [7]. These projects contain high-quality research that benefits from rapid dissemination and are not always published elsewhere. Since the DNP collection was established in 2017 projects have been downloaded over 93,000 times by users around the globe and over 30,000 of those downloads have occurred in the past year. The goal of this project was to explore how linking the Open Access (OA) repository DNP project collection to the library discovery system might expand readership.

## CONTENT MAPPING

The content integration project focused on three phases–extracting relevant metadata from the Digital Commons IR, adding content to library services platform and discovery system, and assessing usage. The project team began by exploring the Ex Libris documentation on Managing Import Profiles [8] and ultimately selected the Repository type import profile as the most effective method for content integration. Unlike other options, the Repository type import profile would allow the team to add content directly into Alma and retain control over item level records. The Himmelfarb Digital Commons IR allows librarians to export collection level metadata via a content inventory report, but at this time there is not an option to export content into any specific metadata schema. MARC 21 bibliographic records are needed for the Repository type import profile and therefore it was necessary for the team to map harvested metadata to the MARC 21 format. The project ultimately created an XLSX template file with 30 MARC 21 fields that could be used for batch adding content from the IR collection to the library services platform and discovery system.

## SUMMARY AND LOOKING FORWARD

In the fall of 2021, the project team successfully batch added 89 bibliography records to the Himmelfarb instances of Alma and Primo VE using the Repository type import profile and the templated spreadsheet. Librarians have also created a DNP project Special Collection in Primo VE [9] and items from this collection have received over 600 link resolver requests since August 2021. In June 2022 the team used the pre-established workflow to add another 15 DNP projects to the library catalog and usage of these items continues. The team now has an effective workflow for integrating collections from Digital Commons into Alma and Primo VE which provides greater control over records than previously available with the OAI feed. Looking forward, there is now a need for more research into the benefits and problems associated with including OA grey literature from an IR in a library discovery system. There is likewise a need to continue to explore the user experience in finding and using this content. The project team will explore future questions such as: what role, if any, do librarians ultimately play in helping to improve readership for institution-specific content, and what emerging technologies might change this landscape?
